# Microstrip Copper Nanowires Antenna Array for Connected Microwave Liquid Sensors

**DOI:** 10.3390/s23073750

**Published:** 2023-04-05

**Authors:** Emanuele Cardillo, Francesco Tavella, Claudio Ampelli

**Affiliations:** 1 Department of Engineering, University of Messina, 98166 Messina, Italy; 2 Department of Chemical, Biological, Pharmaceutical and Environmental Sciences, University of Messina, 98166 Messina, Italy

**Keywords:** antennas, ethanol detection, fifth Generation (5G), microwave electronics, nanowires, smart liquid sensor

## Abstract

In this contribution, a 25 GHz planar antenna, designed and realized in microstrip technology, is exploited as a lightweight and compact liquid sensor. The high working frequency allows minimization of the sensor dimension. Moreover, particular attention was paid to keeping the design cost low. Indeed, the frequency of 25 GHz is widely exploited for many applications, e.g., up to the last decade concerning radars and, recently, 5G technology. Available commercial antennas allowed minimization of the effort that is usually required to design the microstrip sensor. The antenna was in-house realized, and the microstrip Cu conductor was modified through controlled anodic oxidation in order to enhance the sensing features. The sensor capability of detecting the presence and concentration of ethanol in water was experimentally demonstrated. In detail, a sensitivity of 0.21 kHz/(mg/L) and an average quality factor of 117 were achieved in a very compact size, i.e., 18 mm × 19 mm, and in a cost-effective way. As a matter of fact, the availability of devices able to collect data and then to send the related information wirelessly to a remote receiver represents a key feature for the next generation of connected smart sensors.

## 1. Introduction

Microwave liquid sensors are attracting a huge interest in the fields of industrial oil pipelines, fuel systems, food safety, and medicine [1,2].

The success of these technologies lies in the peculiar characteristics of the microwave circuits, mainly in terms of low cost, easy integration with traditional electronic systems and compact dimensions, i.e., the higher the frequency the smaller the size [3,4,5,6,7].

Recently, microwave sensors have been widely exploited to evaluate the dielectric characteristics of different types of materials. As an example, in [8], a complementary symmetric S-shaped resonator was used to estimate the relative permittivity, permeability, and the dielectric and magnetic loss tangents of the material under test. In a similar fashion, in [9], a methodology for the rapid design and tuning of complementary split-ring resonators was proposed with the aim to design microwave sensors smartly for industrial applications. The procedure was validated by measuring the shift in the resonance frequency generated by the material under test.

On the other hand, a recent example of microwave sensors exploited to detect the concentration of saline solution is reported in [1]. In [1], the authors designed an 11 GHz circular resonator with a coupling probe as an advanced sensing system. The measured changes in the resonator operating frequency enabled the detection of concentration changes in the liquid. Microwave sensors are also widely exploited for the dielectric characterization of liquids, whereby an accurate measurement of the complex permittivity is very important in scientific research and industrial applications [10,11,12,13].

A seemingly different topic concerns the field of advanced communication circuits, and in detail 5G and 6G technologies. As a matter of fact, in recent years, a strong correlation between advanced communications and microwave sensors is emerging due to the need to design advanced smart sensors, not only able to measure the parameters of interest but also to collect all the required data and send them to a remote receiver by exploiting the novel paradigm of 5G and, in the near future, 6G technology [14,15]. To this end, very recent contributions focused on microwave antennas exploited as gas or liquid sensors are reported in literature [16,17,18,19,20,21]. The topic is very interesting because employing an antenna as the sensing element enables the design of smart connected sensors directly able to send the measured data. Usually, in a microwave communication system, the antenna is the larger element [22,23,24,25]. To this aim, exploiting the antenna itself as the sensing element allows a reduction of the total system size and a high integrated module to be obtained by incorporating the sensor and the communication device on the same substrate.

The choice of 25 GHz as the antenna working frequency is not casual. Indeed, this frequency falls within the 5G high bandwidth (24–40 GHz) that offers high communication speeds in a relatively small coverage radius and, therefore, is perfectly usable in the recent and future smart sensors.

Moreover, it is worth noting that the UWB industrial, scientific and medical (ISM) band (21.65–26.65 GHz), used in legacy automotive short-range radar sensors, was phased out after 1 January 2022 due to spectrum regulations by the European Telecommunications Standards Institute (ETSI) and the Federal Communications Commission (FCC). Currently, the 24.05–27 GHz band can be only used for tank level probing radar equipment, whereas the automotive band has been limited to the range 24.05 to 24.25 GHz. This made the 24 GHz ISM band unattractive for new radar implementations, despite the great number of related research contributions and commercial solutions.

It is worth noting that the mentioned 5G bands are subject to further limitations related to different parameters, e.g., duty cycle and power levels. As a consequence, the proposed system is not required to transmit while the antenna is detecting the liquid concentration. These tasks can be performed in two separate steps, thus first detecting the liquid concentration, and then transmitting the row or elaborated data. This operating mode allows the change of the main signal parameters during the transmission time, according to the current regulation.

Moreover, the data transmission can be performed without the presence of a liquid, thus avoiding the signal attenuation due to the liquid losses and maximizing the maximum transmitter range.

The choice of 25 GHz allows the re-use of already existent antenna projects, thus drastically lowering the design costs and the know-how required to design the sensor. Indeed, it is well known that continuous great effort is put by the scientific community into the design of novel microwave sensors. Moreover, most of the papers concerning antenna-based sensors exploit lower frequency with a related larger form factor. In this contribution, a 25 GHz microstrip antenna has been designed and realized with in-house facilities. It has been used as a low-cost sensor to detect the presence and concentration of ethanol in water.

Since the proposed sensor uses an antenna as the sensing element, it can be exploited as a part of smart connected sensor networks directly able to send the measured data, thus providing a high integrated module incorporating both the sensor and the antenna on the same substrate.

To enable the sensing properties of the antenna, its surface was modified through controlled anodic oxidation of the Cu conductor. Essentially the substrate was anodized in a two-electrochemical cell containing ethylene glycol with 0.15 M of KOH, 0.1 M NH_4_F, and 3% vol of D.I. water. This technique allows the direct modification of the surface of the conductor and tailors the morphology of the resulting copper oxide to obtain nanoscale structures, such as needles and wires, with a large increase of the active area of the sensor. Nanostructured copper oxide is already known to be a promising material for sensing applications [26], and anodic oxidation allows the growth of a thin oxide layer directly on the Cu substrate to be obtained.

Detecting ethanol is very interesting; indeed, it is a toxic and carcinogenic volatile organic compound that may have long-term impacts on human health as well as on the ecosystem [27]. On the other hand, it is widely used in many fields, e.g., distilleries, wineries and breweries, chemical production, cosmetics, pharmaceutical, food and beverage processing, and refineries. However, because of its high flammability, it is very important to have an effective detection system [28].

The proposed sensor was tested by varying the concentration of ethanol in water from 0 to 100% (vol), and thereafter measuring the frequency shift of the Scattering (S-) parameter S_11_, by exploiting a network analyzer.

This manuscript is organized as follows. In Section 2 the sensor design and realization are described. The main results arising from experimental analysis and the relative discussion are shown in Section 3. Finally, the conclusive remarks are drawn up in Section 4.

## 2. Antenna Sensor Design and Realization

The antenna array was designed according to the simplified formulation described in [29]. It is composed by four microstrip patches whereby the main parameters of interest are the patch width W and length L. For an efficient radiator, a practical width that leads to good radiation efficiencies is calculated as in

(1) W=2ϵr+12frϵ0+1

where $\epsilon_{r}$ is the substrate permittivity, $f_{r}$ is the central operating frequency, and $\epsilon_{0}$ is the value of the absolute permittivity of vacuum. To calculate the proper patch length, whose typical values for microstrip antennas vary between 0.47 $\lambda_{d}$ and 0.49 $\lambda_{d}$, where $\lambda_{d}$ is the wavelength in the dielectric, it is necessary to calculate the effective permittivity $\epsilon_{reff}$. For Wh>1, where h is the dielectric thickness, it can be computed as in

(2) ϵreff=ϵr+12+ϵr−121+12hW.

Thereafter, the patch length can be calculated as reported in (3).

(3) $$L=\frac{1}{2f_{r}\sqrt{\epsilon_{reff}}\sqrt{\epsilon_{0}\mu_{0}}}-2∆L$$

where the term ∆L is a correction factor due to the fringing effects. Indeed, the electrical length of the microstrip patch looks greater than its physical dimensions, as a function of the effective dielectric constant and the width-to-height ratio.

A known practical approximate relation for the normalized extension of the length is reported in

(4) ∆L=0.412hϵreff+0.3Wh+0.264ϵreff−0.258Wh+0.8.

The patch parameters were calculated for the RO4350B hydrocarbon ceramic laminates substrate by Rogers Corporation, which is characterized by a dielectric constant equal to 3.48, a dissipation factor equal to 0.0037, and a dielectric thickness of 254 mm, all provided by the manufacturer at the frequency of 10 GHz.

**Figure 1:**
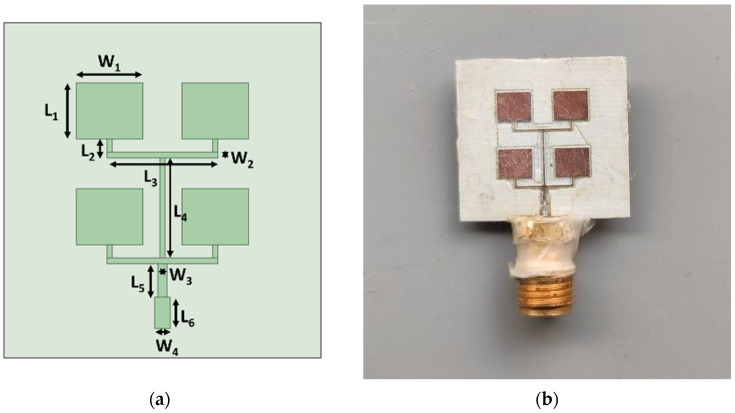
(**a**) Layout and (**b**) picture of the antenna array.

The patch parameters were simulated by exploiting the Ansys Electromagnetic Suite High-Frequency Structure Simulator (HFSS). In order to obtain a real impedance close to 50 Ω, the resulting microstrip antenna array was optimized. Thereafter, the resulting impedance was matched by exploiting a λ4 transformer. Finally, the antenna was realized by means of in-house facilities, i.e., by exploiting the microwave circuit board plotter LPKF ProtoMat S103. In Figure 1a,b, the layout and a picture of the antenna array are shown, and the related main layout parameters are reported in Table 1. The total size of the microwave antenna sensor is 18 mm × 19 mm.

**Figure 2:**
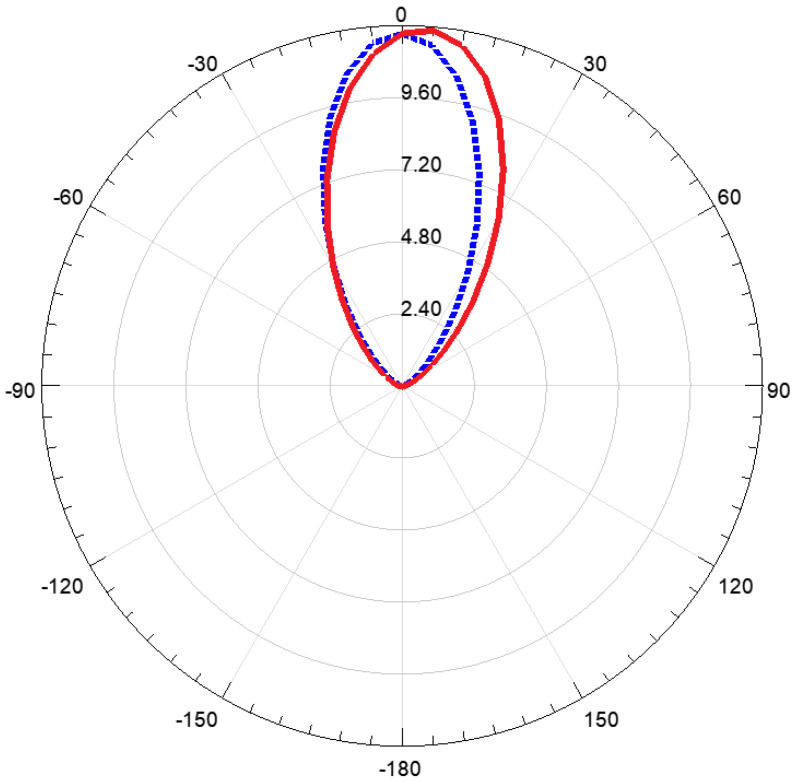
Antenna radiation pattern in the horizontal (dotted blue) and vertical (solid red) planes.

The simulated radiation pattern of the antenna for both the horizontal and vertical planes is reported in Figure 2. The maximum gain is equal to 11.7 dBi.

Employing an antenna array, instead of a single patch element, allows the increase of the antenna gain. This feature does not improve the sensor performance but allows the effectively use of the antenna to transmit the detected data. A four-element array was considered a good tradeoff between the antenna gain (11.7 dBi) and the total sensor dimension.

The antenna surface was modified through controlled anodic oxidation of the Cu conductor [30]. Essentially, the substrate was anodized in a two-electrochemical cell containing ethylene glycol with 0.15 M of KOH, 0.1 M NH_4_F, and 3% vol of D.I. water.

**Figure 3:**
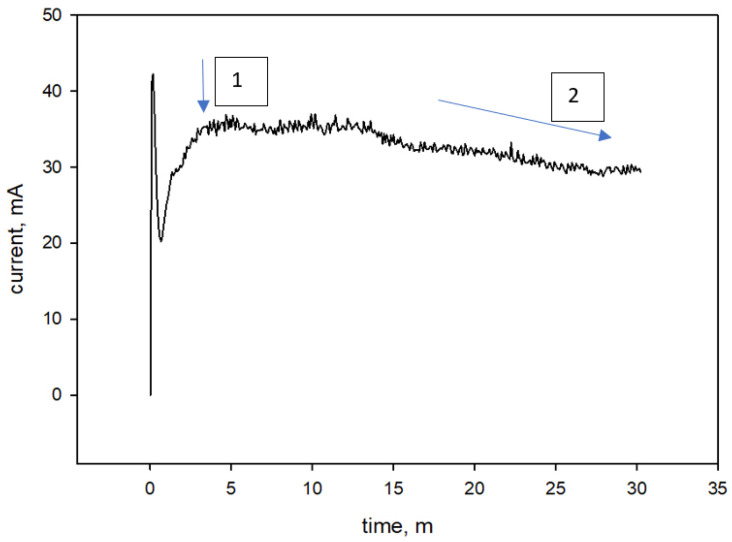
Current vs. time current profile obtained during the anodization procedure. The barrier oxide layer formation is highlighted by the arrow n. 1, whereas the point whereby the current starts to gradually diminish due to the growth of the oxide layer nanostructure is indicated by the arrow n. 2.

The anodization was carried out at 30 V for 30 min. The prepared sample was later rinsed in water and dried in air. The essence of the method can be described as a reconstruction of a thin CuOx layer that occurs under the application of a constant voltage in the presence of fluoride-based electrolytes. Figure 3 shows the time/current profile obtained during the anodization procedure of the Cu substrate.

In the first 5 min of anodization, the current increases until a maximum is reached (~36 mA) due to the application of the voltage. This is the step in which the barrier oxide layer formation occurs, and it is indicated in Figure 3 by means of the arrow number 1. Later, the current starts to gradually diminish due to the growth of the oxide layer nanostructure that possesses a higher electrical resistance with respect to the starting metallic layer, and it is indicated in Figure 3 by means of the arrow number 2. The growth of the nanostructure is operated by the equilibrium of two different reactions: from one side the oxide layer grows under the application of the external potential, and, on the other hand, the dissolution operated by the fluorine ions digs the oxide layer, resulting in the creation of the nanowires.

**Figure 4:**
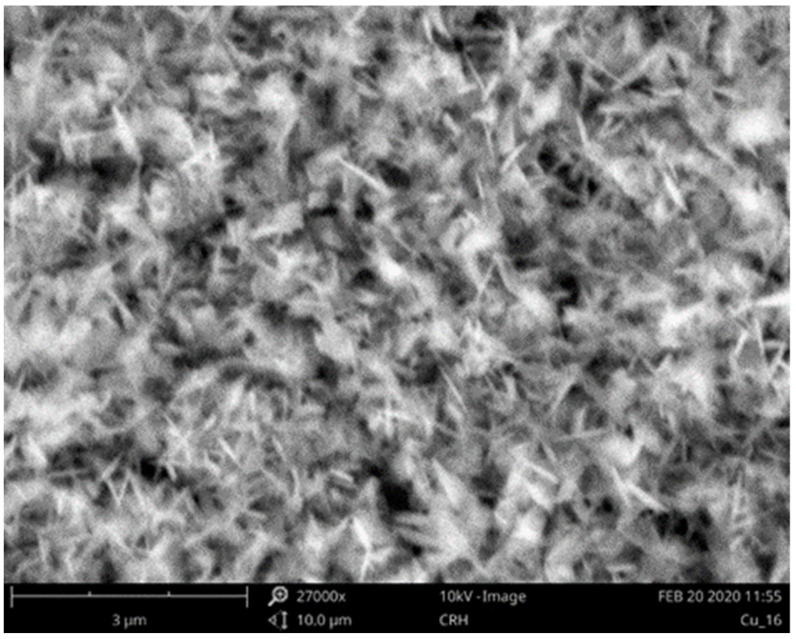
SEM-EDX picture of the Cu microstrip conductor.

Figure 4 shows the SEM-EDX picture of the Cu microstrip conductor, extracted by exploiting the scanning electron microscope Phenom ProX Desktop. It is possible to notice the presence of a well-defined CuOx nanowire structure covering the entire surface of the Cu conductor layer. As many authors report in the literature, CuO is highly selective in the absorption of ethanol and is thus largely used as active material for electrochemical ethanol detection. However, only a few contributions describe how to exploit these materials as microwave sensors [31,32,33,34,35,36]. Using the described method allows high CuOx layer surface area growth directly on the Cu substrate to be obtained. This treatment not only allows the large increase of the surface area of the sensor but also avoids the leaching of the active material from the substrate, like in the case of the classic deposition methodologies, thus resulting in an increase of the overall robustness of the sensor.

The antenna capability to behave like a sensor is based on the different electrical response due to permittivity variations. Indeed, in a microstrip line, the phase velocity $v_{p}$ and the propagation constant β can be expressed as in [37].

(5) $$v_{p}=\frac{c}{\sqrt{\epsilon_{reff}}}$$

(6) $$\beta =k_{0}\sqrt{\epsilon_{reff}}$$

where $k_{0}$ is the propagation constant (wave number) of a plane wave in free space. It is worth noting that $\epsilon_{1}<\epsilon_{reff}<\epsilon_{r}$, where $\epsilon_{1}$ is the permittivity of the material on the top side of the microstrip.

Usually, microstrip lines are surrounded by air, thus $\epsilon_{1}$ is considered very close to 1. When the antenna is placed in a solution of only water, $\epsilon_{1}$ becomes almost equal to 80 at room temperature and for very low frequencies. It is worth noting that, due to relaxation processes, the higher the frequency the lower the permittivity.

The presence of a liquid with a different permittivity will modify the total value of $\epsilon_{1}$, as for the case of the ethanol, whose permittivity is equal to 24.55. This change can be detected by exploiting the related shift of the antenna resonance frequency, which can be highlighted my measuring the related scattering matrix. S-parameters provide a complete description of the N-port network. Since antennas are one-port networks, the only parameter of interest is the S_11_. As discussed in the Introduction, this contribution investigates the possibility of exploiting already designed antennas in order to decrease the design cost of the sensor. As a matter of fact, the typical employed geometries for microstrip resonators require an advanced knowledge of microwave engineering topics.

## 3. Experimental Analysis

**Figure 5:**
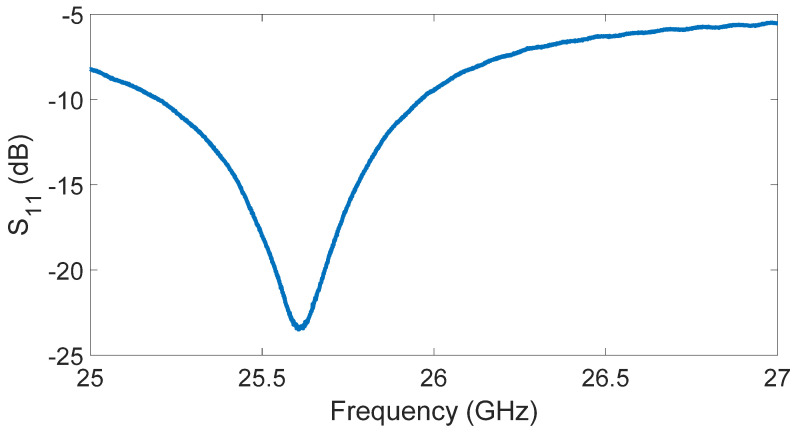
S_11_ of the antenna in presence of water.

The performance of the microwave liquid sensor was evaluated using a network analyzer, in detail an Agilent E8364A (0.045–50 GHz). The S_11_ of the antenna immersed in water is shown in Figure 5.

For the sake of completeness, a typical shift between the original antenna design frequency and the measured one was observed after the anodization procedure. The central antenna operating frequency was equal to 25.61 GHz. The data were measured after the instrument calibration, which was required to correct systematic and drift errors and to shift the true measurement reference planes as close as possible to the device under test. The calibration procedure was performed by employing a 3.5 mm 80050Q calibration kit by Maury Microwaves.

**Figure 6:**
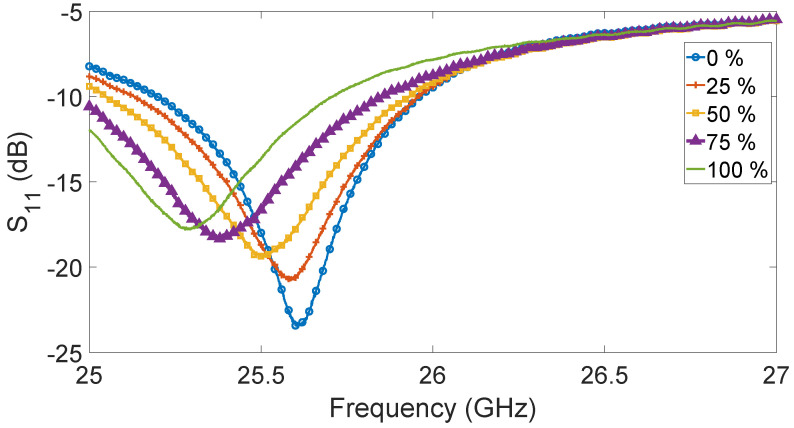
S_11_ of the antenna immersed in a solution of ethanol and water with different concentrations: 0% (blue solid line with circles), 25% (orange solid line with plus signs), 50% (yellow solid line with squares), 75% (purple solid line with triangles), and 100% (green solid line without markers).

Different measurements were performed by changing the concentration of ethanol in water. All the measurements were performed at the temperature of 20 °C. In detail, Figure 6 shows the changes in the S_11_ parameter when the concentration varied from 0 to 100% with a 25% step.

From Figure 6, it is possible to observe the evident S_11_ shift due to different ethanol concentrations.

With the increasing of the concentration, a reduced S_11_ module can be observed. This behavior might be expected because the antenna was working at an operating point different from the designed one.

The main sensing effects are only due to the upper conductor interaction with the liquid, whereas the bottom conductor represents the well-known ground reference plane typical of microstrip circuits and in detail of microstrip antennas.

**Figure 7:**
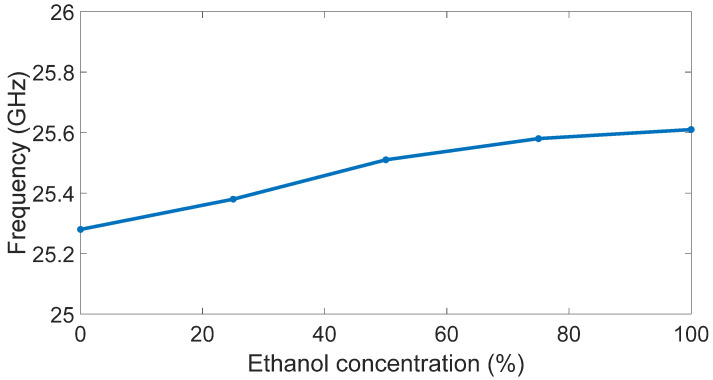
Antenna resonance frequencies versus ethanol concentration.

The different resonance frequencies are better highlighted in Figure 7, where they are represented for different ethanol concentrations.

**Figure 8:**
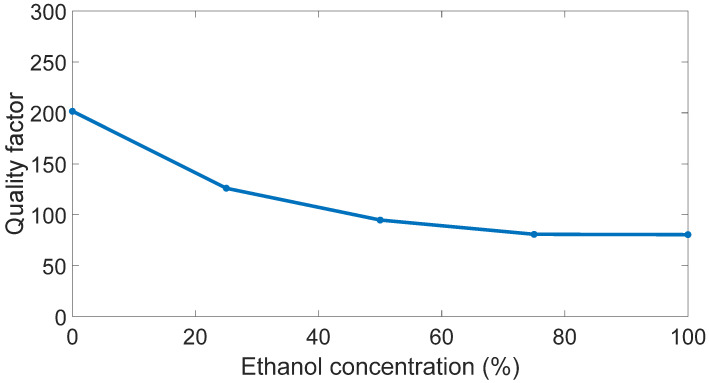
Antenna quality factor versus ethanol concentration.

For each different ethanol concentration, the quality factor of the sensor was calculated by considering the ratio between the central operating frequency and the −3 dB bandwidth. An average value of 117 was estimated, and the different values are reported in Figure 8.

**Figure 9:**
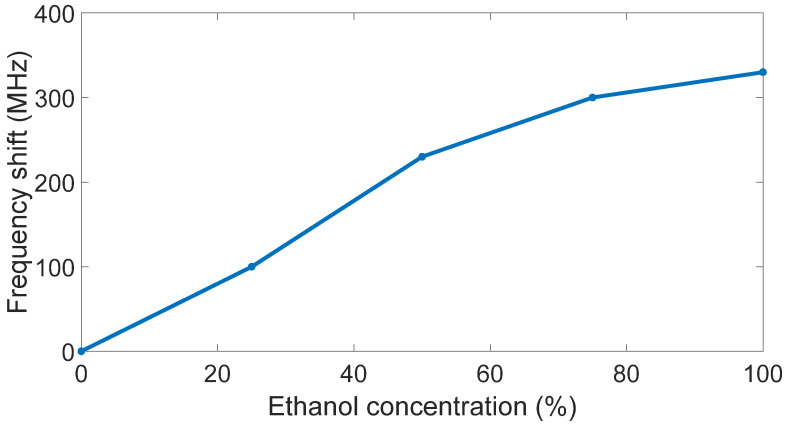
Antenna frequency shift versus ethanol concentration.

It is worth highlighting the frequency shifts related to the case where the sensor was immersed only in water. The different values of frequency shifts are reported in Figure 9.

A sensitivity equal to 0.21 kHz/(mg/L) was estimated. Both the sensitivity and the quality factor of the sensor are in line with the average values reported in the literature [16,17,18,19,20,21]. However, this result was achieved with a small form factor, i.e., 18 mm × 19 mm, and minimizing the design cost by exploiting an already available K-band antenna. As a final feature, this sensor enabled a direct integration in a sensor network where the sensor node was already and intrinsically equipped by the antenna element.

## 4. Conclusions

In this contribution, a microwave antenna working in the K-band has been designed and tested as a liquid sensor. In detail, it was exploited to detect the concentration of ethanol in water. This task was accomplished by maintaining very compact dimensions and a low cost, which are key requirements for a sensor. Moreover, since the antenna design procedure can be considered a well-consolidated task, the sensor design is very straightforward and thus reproducible by those researchers interested on this topic.

To this aim, an already available antenna was used, thus exploiting the reusability of components along with keeping the design cost low.

The results of this work can be exploited to realize compact sensors, which are able to carry out the dual function of being the sensing element and the radiating element required for data transmission.

## Figures and Tables

**Table 1:** Patch array geometrical parameters.

Element ID	Value
L_1_	3.20 mm
L_2_	1.05 mm
L_3_	6.30 mm
L_4_	5.70 mm
L_5_	1.90 mm
L_6_	1.80 mm
W_1_	3.80 mm
W_2_	300.00 μm
W_3_	500.00 μm
W_4_	870.00 μm

## Data Availability

Not applicable.
